# Madhouses before A.D. 1846

**Published:** 1918-03-02

**Authors:** 


### PAPERS ON MADNESS.

### I.—MADHOUSES BEFORE A.D. 1846.

Parliament—how naturally do one's thoughts revert to Parliament when one speaks of madness! There was once in this country a Parliament called the Mad Parliament, and there is now a Parliament that may hereafter be called the Fatuous Parliament—Parliament in 1828 decreed that from thenceforth no madman should be placed under control in what was then for the first time called an asylum except upon the certificates of two medical practitioners. Unlike most of the enactments of Parliament, this enactment was beneficial. It put a stop once for all to the common practice of sending anyone whose absence was desired, or over whom it was desired to get control, to a madhouse without any formality other than asking the keeper of the madhouse to receive the patient, and paying him as long as he kept the patient. This had been the practice before 1828, and many abuses had clustered round the practice. Sane persons, and persons who were known to be sane, against whom no allegation of madness could have been sustained for a moment, were sent to madhouses to get them out of the way. The madhouses were under no sort of control. They could receive whom they pleased, keep their patients as long as they pleased, and treat them as they pleased. Not until 1828 was any licence required for keeping a madhouse, and when it was at length enacted that no one should keep a madhouse without a licence, it was at the same time enacted that the licensing body, the College of Physicians, should grant a licence to anyone who should apply for one. The College had no power to inquire into the fitness of the licensee to hold a licence for such a purpose, nor into the fitness for the purpose for which they were to be used of the premises he occupied. The College had, it is true, the power, and even was charged with the duty, of inspecting the madhouses in the metropolis and for a certan distance round it, and might even withdraw the licence of a house that was badly managed; but the College was compelled to grant licences to all who should apply for the same, and when it cancelled a licence, the licensee immediately applied for another, which the College could not refuse to grant, so that the terror of the withdrawal of his licence was not very effectual in restraining the licensee from evil practices if he felt inclined to resort to them. And in some cases—in many cases, in fact—he was inclined to resort to them, the more so perhaps because in many cases it paid him to resort to them. It is unlikely that the relatives of a patient or the party who sent a patient to a madhouse, who need not be a relative, but might be anyone who had an interest in getting the patient out of the way, ever said to the keeper of the madhouse in plain terms, "I want this person murdered, and I will give you so much down on proof of his death"; but a nod is as good as a wink to a blind horse, and the keepers of the madhouses were not blind, at least they were not blind to their own interests, nor to the powers which the wisdom of Parliament permitted them to exercise. King John's instructions to Hubert show us how a request to commit a murder may be conveyed plainly enough without any mention of such an ugly word as murder, and that patients were murdered in madhouses before 1828 and afterwards, there is ample evidence to show.

Short of actual, wilful, deliberate murder, cruelty and neglect towards the unfortunate patients were rampant, and it is to be remembered that under the system permitted by the wisdom of the Legislature to exist and continue some of the patients in madhouses were not mad, at any rate when they were first captured and taken to these institutions. There was no particular reason why they should be. No certificate, no formality of any kind, was required by law or was employed in practice. Anyone who could pay for the maintenance of a person in a madhouse could send to a madhouse anyone he pleased, and there was no one to say him nay. Nor was there any means of obtaining the liberation of a patient. As long as payments were continued, the patient, mad or not mad, was detained. When the payments ceased, the patient, mad or not mad, could be turned into the street.

In these circumstances the keeping of madhouses fell into the hands of persons who were in many cases utterly unscrupulous, and who administered their institutions on the simple principle of spending upon them as little as possible, and making out of them the maximum of profit. The food the patients received was in quantity the very least that would hold body and soul together. Of clothing many of them had none at all. They lay naked upon straw. Their accommodation was always comfortless and often loathsome, and their treatment in other respects was abominable. Even at Bethlem, which was a Royal Hospital, a quasi-charitable institution in which the managers had no pecuniary interest, a place that set up a standard to the height of which few other madhouses attained, a place that was a common show which anyone could visit and inspect for the trifling payment of sixpence, and was, in fact, an amusing Sunday resort for the citizens of London, a place, therefore, in which anything that was done was done more or less under the public eye, the keepers perambulated the wards with whips stuck in their belts, and never hesitated to use these instruments upon the patients when occasion seemed to require it. It is to be remembered that the language, conduct, and demeanour of many mad persons are exasperating in the extreme, and are often prompted by a steady and rooted malignity that renders them additionally exasperating. It is to be remembered also that one of the most conspicuous features of every kind of madness is the absorbing and callous selfishness of the patient; and selfishness in other people is always exasperating. Even in these days, when nurses, both female and male, in asylums are carefully and

**PAPERS ON MADNESS—(*continued*).**

assiduously trained to act towards their patients with kindness, forbearance, and consideration; when the atmosphere of the asylum is an atmosphere of humanity; and when a carefully-graded hierarchy of officials ensures constant and vigilant supervision; acts of cruelty are occasionally perpetrated; but before the Lunacy Act, 1828, cruelty was the rule, and was systematic. Among primitive peoples madness is attributed to possession by an evil spirit, and those who are familiar with the selfishness of nearly all the insane, with the impishness of many, and with the studied malignity of not a few, cannot wonder that this opinion should prevail. The treatment of madness, where this notion of its origin and nature prevails, is naturally adapted to removal of the cause. If an evil spirit inhabits the body, the way to get rid of the evil spirit is to make it as uncomfortable as possible, so that it may become disgusted with its habitation and take its departure. It is considered that to torment and ill-treat the body that contains the evil spirit is equivalent to tormenting and ill-treating the spirit itself; and therefore any cruelty that is inflicted upon the patient is a justifiable, and even a necessary, mode of treatment, and the mode best calculated to ensure the expulsion of the cause and the recovery of the patient. Hence, not only did humanity exert no check upon the ill-treatment of the madman, but it even dictated and stimulated cruelty towards him. Flogging and ill-treatment of various kinds became a recognised and accepted therapeutic measure, and the practice continued long after the belief had faded. The belief faded, but rags and remnants of it may still be recognised in our estimation and treatment of madness.

Even after the passing of the Lunacy Act, 1846, the Magna Charta of the mad, even after the institution of public asylums under the disinterested administration of public bodies of enlightened men, cruelties that were thought to be necessary and even beneficial were continued. Before this Act was passed, chains, shackles, handcuffs, iron belts, iron collars, even muzzles, were in routine employment in the restraint of mad people, and whips and floggings and other modes of physical violence were regarded as legitimate and proper modes of treatment. Even a king, when mad, was subjected to physical violence by his keepers. Not until Conolly and Samuel Tuke in this country, and Pinel in France, had demonstrated by actual experiment that it was not only possible, but beneficial, to treat madness without brutality were these brutal methods abandoned, and even after their futility had been demonstrated for generations, even after the alternative system of humanity had been shown to be practicable, the brutal methods were abandoned slowly, reluctantly, and piecemeal.

